# Identity of *MMP1* and its effects on tumor progression in head and neck squamous cell carcinoma

**DOI:** 10.1002/cam4.4623

**Published:** 2022-04-14

**Authors:** Gehou Zhang, Tieqi Li, Guolin Tan, Yexun Song, Qian Liu, Kai Wang, Jingang Ai, Zheng Zhou, Wei Li

**Affiliations:** ^1^ Department of Otolaryngology‐Head Neck Surgery Third Xiangya Hospital of Central South University Changsha Hunan Province China; ^2^ Department of Otolaryngology‐Head Neck Surgery The First Affiliated Hospital of Shaoyang University Shaoyang China

**Keywords:** differentially expressed genes, head and neck squamous cell carcinoma, hub genes, integrated bioinformatics analysis, *MMP1*

## Abstract

**Background:**

Head and neck squamous cell carcinoma (HNSCC) is the sixth most common malignant tumor worldwide with high morbidity and mortality. However, the diagnosis and molecular mechanisms of HNSCC remains poor.

**Methods:**

Robust rank aggregation method was performed to excavate the differentially expressed genes (DEGs) in five datasets (GSE6631, GSE13601, GSE23036, GSE30784, GSE107591) from the Gene Expression Omnibus. Search Tool for the Retrieval of Interacting Genes (STRING) database extracted hub genes from the protein‐protein interaction network. The expression of the hub genes was validated using expression profile from The Cancer Genome Atlas and Oncomine database. The module analysis and disease‐free survival analysis of the hub genes were analyzed by Cytoscape and the Kaplan‐Meier curve, respectively. The expression of hub genes was verified in clinical specimens. The functions of *MMP1* which is most important in hub genes were explored in vitro and in vivo.

**Results:**

Totally, 235 DEGs were identified in the present study which consists of 103 up‐regulated and 132 down‐regulated genes which were significantly enriched in the molecular function of calcium ion binding followed in the biological process of skin development. The mainly enriched pathways were ECM (extracellular matrix)‐receptor interaction (hsa04512) and protein digestion and absorption (hsa04974). Six hub genes were screened out which showed dramatically increased expression in HNSCC samples compared with normal samples, including *COL4A1*, *MMP1*, *PLAU*, *RBP1*, *SEMA3C*, and *COL4A2*. These hub genes all showed worse disease‐free survival with higher expression and were up‐regulated in HNSCC clinical samples. *MMP1* was proved to promote cell growth, migration, and phosphorylation of AKT in vitro and to promote liver metastasis in vivo.

**Conclusion:**

Bioinformatics analysis identified six key genes in HNSCC. Of these, *MMP1* is the most likely biomarker. It activates the AKT pathway and promotes tumor progression.

## Graphical abstract

MMP1 is the most potential biomarker in head and neck squamous cell carcinoma according to our bioinformatic analysis. This figure showed the effect of MMP1 in vitro.

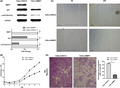

## 1 INTRODUCTION

Head and neck squamous cell carcinoma (HNSCC) is the most common malignant tumor of the head and neck in the world.^1^ Although the level of surgery and the efficacy of chemotherapy drugs have improved significantly in the past half century, the 5‐year survival rate of HNSCC has not improved significantly, and the survival rate in patients diagnosed with advanced disease is only about 50%.^2^ In addition, although increasing numbers of studies have focused on the mechanism of HNSCC formation and progression, the molecular mechanism of HNSCC is still unclear.^3^ Therefore, exploring reliable and effective markers and the hub genes which are essential to the discovery of molecular mechanisms of HNSCC is urgently needed.

With the development of next generation sequencing (NGS) and chip technology, the datasets produced by these NGS and chip have been applied to exploring amounts of molecular biomarkers in different cancers,^4^, ^5^ especially for the Gene Expression Omnibus (GEO) and The Cancer Genome Atlas (TCGA) databases.^6^, ^7^ Although it can rapidly screen out the differential expressed genes (DEGs) which have important effects on cancer progression, it may cause biased and unreliable results because of the high expenses and limited sample tissues in individual experiments. Therefore, in order to obtain accurate and reliable results, amounts of studies have been analyzed by integrated bioinformatics analysis using multiple datasets. So far, various gene chips have been used in many studies to identify key molecular factors in HNSCC and various genes.^8^, ^9^, ^10^ For example, Feng et al screened 1459 differentially expressed lncRNAs and 2381 DEGs in advanced laryngeal squamous cell carcinoma by lncRNA and mRNA integrated microarrays^11^; Recently, *HOTTIP* was found to be a key candidate biomarker by Yin et al and *SERPINE1*, *PLAU*, and *ACTA1* could act as biomarkers in HNSCC detected by Yang et al via integrated bioinformatics analysis.^10^, ^12^ However, there are few reliable diagnostic biomarkers and therapeutic targets for HNSCC. In addition, the involved mechanisms in the development of HNSCC for the hub genes obtained by integrated bioinformatics analysis are not clear. Therefore, it is really urgent to discover the effective molecular biomarkers and the molecular mechanism of HNSCC which will be crucial to the diagnosis and treatment of HNSCC.

In the present study, we performed an integrated bioinformatics analysis to explore the DEGs from five GEO datasets. Furthermore, Gene Ontology (GO) annotation and Kyoto Encyclopedia of Genes and Genomes (KEGG) pathway analyses were performed to assess functional pathways of DEGs. And the hub genes were extracted from a protein‐protein interaction (PPI) network and the expression of the hub genes were validated using expression profile from TCGA and Oncomine database. The Kaplan‐Meier curve of these genes was used to evaluate disease‐free survival of the six hub genes based on TCGA and GTEx in GEPIA. We detected their expression in clinical specimens. Finally, we focused on *MMP1* and confirmed its function in vitro and in vivo.

## 2 MATERIALS AND METHODS

### 2.1 Materials

Human hypopharyngeal squamous cell carcinoma cell, FaDu, was purchased from Suzhou Bei Na Chuanglian Biotechnology Co., Ltd. (#BNCC316798). minimum essential medium (MEM), bicinchoninic acid (BCA) protein assay kit, and SDS‐PAGE gel preparation kit were bought from KeyGEN BioTECH Inc. RNA interference lentivirus with a puromycin resistance gene was bought from GeneChem Inc. (siMMP1 RNA sense was 5’‐CAAGGGAUAACUCUUCUAAdTdT‐3′, and antisense was 5’‐UUAGAAGAGUUAUCCCUUGdTdT‐3’^13^). E.Z.N.A.® miRNA Kit was from Omega Bio‐Tek Inc. ReverTra Ace® qPCR‐RT Master Mix with gDNA Remover and KOD SYBR® qPCR Mix were from TOYOBO Co., Ltd. Primers for quantitative real‐time polymerase chain reaction (qRT‐PCR) were purchased from GeneCopoeia Inc. Phosphatase inhibitor was from CWBIO Inc., polyvinylidene fluoride (PVDF) membrane from Merck Millipore, and bovine serum albumin (BSA) powder from Sigma‐Aldrich. Radio immunoprecipitation assay buffer (RIPA), phenylmethylsulfonyl fluoride (PMSF), AKT inhibitor VII and primary antibodies of AKT (1:1000 dilution), and p‐AKT (Ser473) (1:2000 dilution) were from Beyotime Inc. Primary antibodies of glyceraldehyde‐3‐phosphate dehydrogenase (1:1000 dilution) and MMP1 (1:1000 dilution) were from Proteintech Inc. Horseradish peroxidase‐conjugated goat anti‐rabbit IgG secondary antibody (1:10,000 dilution) was bought from Signalway Antibody Co., Ltd. Ethanol, xylene, and neutral gum were from Sinopharm Chemical Reagent Co., Ltd. HE dye solution set was bought from Wuhan Servicebio Technology Co., Ltd. BALB/C nude mice were bought from Hunan SJA Laboratory Animal Co., Ltd.

### 2.2 Collection of tissue specimens

Carcinoma of tonsil, laryngeal cancer, hypopharyngeal cancer, carcinoma of the buccal mucosa, tongue cancer tissues, and their adjacent normal tissues (*n* = 3) were obtained from patients in the Third Xiangya Hospital (Changsha, People's Republic of China).

### 2.3 Data acquisition and preprocessing

All the five GEO datasets were downloaded from GEO (https://www.ncbi.nlm.nih.gov/geo/) using GEO query described by Sean and Meltzer,^14^ including GSE6631, GSE13601, GSE23036, GSE30784, GSE107591. The detailed information of all the five GEO datasets is listed in Table 1. The raw microarray data of expression files were normalized and log2‐transformed. DEGs were identified by the Bioconductor Limma package and then robust rank aggregation method was used to integrate and rank all of the DEGs from five GEO datasets. In addition, the edge R package was used to screen DEGs with thresholds of |log2‐fold‐change[FC]|>1 and the thresholds of the adjusted *p* < 0.05.

**TABLE 1:** The detailed information of the five GEO datasets

Dataset	Numbers of samples (tumor/normal)	Array types	Experiment type	Origin
Kuriakose MA et al., GSE6631	22/22	Affymetrix Human Genome U95 Version 2.0 Array	mRNA	Cell Mol Life Sci
Estilo CL et al., GSE13601	29/29	Affymetrix Human Genome U95 Version 2.0 Array	mRNA	BMC Cancer
Pavón MA et al., GSE23036	34/34	Affymetrix Human Genome U133A 2.0 Array	mRNA	Carcinogenesis
Chen C et al., GSE30784	167/62	Affymetrix Human Genome U133 Plus 2.0 Array	mRNA	Cancer Epidemiol Biomarkers Prev
Verduci L et al., GSE107591	24/23	Affymetrix Human Gene 1.0 ST Array	mRNA	Genome Biol

Abbreviation: GEO, Gene Expression Omnibus.

### 2.4 GO analysis and KEGG pathway analysis

The DEGs from GEO database were analyzed by an online program Database for annotation, visualization, and integrated discovery (DAVID) (http://david.abcc. ncifcrf.gov/).^15^ The GOchord R package and DAVID database were used to perform GO analysis and KEGG pathway maps with cut‐off *p* < 0.05, respectively.^16^

### 2.5 PPI network construction

The PPI network was performed by the Search Tool for the Retrieval of Interacting Genes (STRING) (https://string‐db.org/) for the DEGs identified in the five GEO datasets. The hub genes were identified by Cytoscape and modules of hub genes from the PPI network were screened by the Molecular Complex Detection (MCODE).^17^

### 2.6 Hub genes analysis

The seed genes in modules with the most connectivity referred to hub genes and TCGA data was used to perform validation using GEPIA database.^18^ The analysis for expression level of hub genes between HNSCC samples (*n* = 519) and normal samples (*n* = 44) was based on GTEx data in GEPIA from TCGA. Oncomine database was used to further analyze the expression level of hub genes with clinical traits.^19^ Logrank value *p* < 0.05 was considered to be statistically significant.

### 2.7 Cell culture and infection of lentivirus

FaDu cell was cultured in MEM containing 10% fetal calf serum (FBS) at 37°C in an incubator with 5% CO_2_. SiMMP1 lentivirus can stably downregulate the expression of *MMP1*. The controlling lentivirus (CON313) carries only puromycin resistance gene but no siRNA. Two kinds of lentivirus infected FaDu cells, respectively. Cells were infected by lentivirus at MOI = 100 with HitransG P virus infection reagent for 16 h. Two μg/ml puromycin was used to select steadily infected cells. FaDu‐CON313 was infected with controlling lentivirus, while FaDu‐siMMP1 with lentivirus containing siMMP1 RNA.

### 2.8 QRT‐PCR

RNA of tissues was isolated by E.Z.N.A.® miRNA Kit. Concentration of RNA was tested by Thermo® NanoDrop 2000. After reverse transcription, Roche LightCycler® 480 detected amplification for qRT‐PCR. The expression of mRNAs was normalized by *ACTB* (beta‐actin).

### 2.9 Western blotting (WB)

Cells and tissues were lysed by RIPA containing PMSF and phosphatase inhibitor. Concentration of protein was tested by BCA protein assay kit. 10% SDS‐PAGE gels were prepared for separating 10–30 μg total protein. The protein was transferred to the PVDF membrane, blocked in 5% BSA for 1 h, and incubated in primary antibodies at 4°C overnight. Then, the membrane was incubated in secondary antibody for 1 h. Enhanced chemiluminescence reaction was applied to detect the protein expression.

### 2.10 Cell growth curve

3 × 10^4^ FaDu‐CON313 and FaDu‐siMMP1 cells per well were, respectively, seeded in a 24‐well plate. Three wells of cells were each digested with pancreatin and counted under 10 × 10 microscope for 7 consecutive days.

### 2.11 Wound‐healing assay

4 × 10^5^ cells per well were seeded in a 24‐well plate and cultured in MEM containing 10% FBS at 37°C in an incubator with 5% CO_2_ for 24 h. Then, 200‐μl pipet tips were used to scrape them. Cells were then cultured in MEM containing 1% FBS. Images of wounds were captured after 0 and 24 h.

### 2.12 Transwell migration assay

5 × 10^5^ cells per well were seeded in transwell chambers with 8.0 μm pore membrane. 100 μl MEM without FBS was in the upper chambers and 500 μl MEM with 10% FBS was in the lower wells. After being cultured at 37°C for 24 or 48 h, cells were fixed by methanol and dyed by crystal violet. All figures were captured under 10 × 10 microscope. Five fields were collected for each chamber, and 3 chambers for each group.

### 2.13 AKT inhibiting and colony formation assay

AKT inhibitor VIII (AKTi) could inhibit Akt1, Akt2, and Akt3 activity. The final concentration of AKTi in the experimental group was 10 μM. For wound‐healing assay, cells were pretreated with AKTi for 12 h before being scraped. For transwell migration assay, the upper chambers were added with AKTi. For colony formation assay, 2 × 10^3^ cells per well were seeded in a 24‐well plate. Cells were treated with 10 μM AKTi for 12 h. After being totally cultured for a week, cells were fixed by methanol and dyed by crystal violet.

### 2.14 In vivo subcutaneous tumor model

200 μl PBS dissolving 2 × 10^6^ FaDu‐CON313 or FaDu‐siMMP1 cells were respectively injected into two male 4–6‐week‐old BALB/C nude mice under the skin of armpit to obtain tumor tissues. These tumors were cut into about 1 mm^3^ masses of tissue. The other eight male 4–6‐week‐old BALB/C nude mice were randomly divided into two groups (*n* = 4). Tumor tissues derived from FaDu‐CON313 and FaDu‐siMMP1 were, respectively, buried under dorsal skin. These eight nude mice were executed after 4 weeks to compare volume of tumors and anatomized to estimate situation of metastasis. These nude mice were euthanized through cervical dislocation.

### 2.15 HE staining

Paraffin sections were dewaxed by xylene and ethanol and rinsed with water. Sections were stained by hematoxylin solution, differentiated by hematoxylin differentiation solution, and blued by hematoxylin Scott’s tap bluing. Then, the sections were dyed with eosin. Finally, sections were dehydrated by ethanol and xylene, and observed with microscope inspection.

### 2.16 Statistical analysis

All statistical analyses in the present study were calculated using SPSS 19.0 (SPSS Inc.). Cells of migration, density of WB bands, and area of colonies were calculated by ImageJ 1.52a. All bar charts and cell growth curve were drawn by GraphPad Prism 5. All data are presented as mean ± standard deviation (SD). Statistical significance between two groups was evaluated by Student's *t*‐test between two groups. *p* < 0.05 was statistically significant.

## 3 RESULTS

### 3.1 Identification of DEGs in HNSCC among five GEO datasets

**FIGURE 1:**
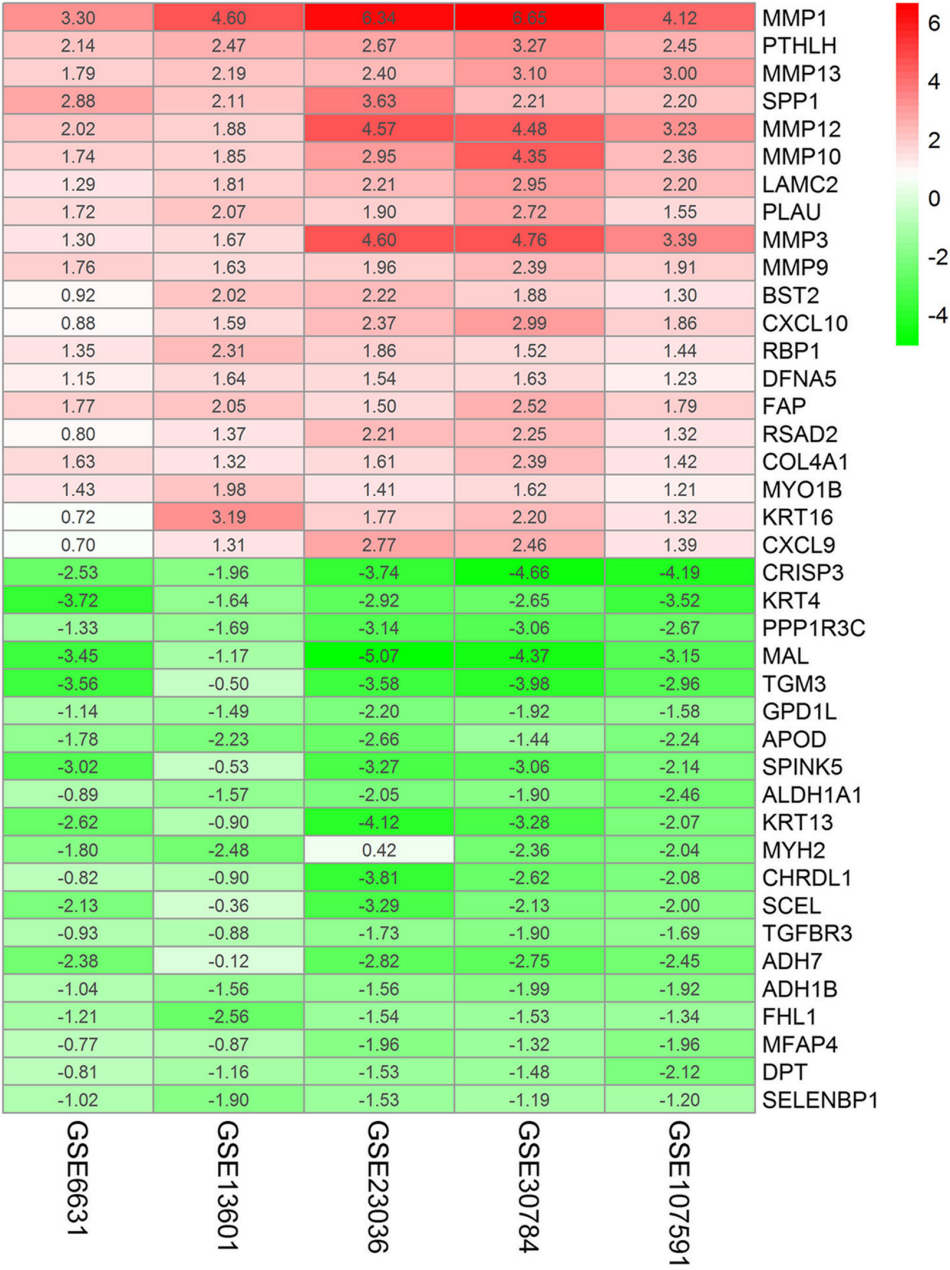
Identification of DEGs among each GEO dataset. The expression heat map of the 20 robust DEGs using the RRA method. Red indicates overexpressed genes in HNSCC and green for underexpressed. DEGs, differentially expressed genes; GEO, Gene Expression Omnibus; HNSCC, head and neck squamous cell carcinoma

In order to explore the DEGs in HNSCC, five GEO datasets containing 170 normal samples and 276 HNSCC samples were downloaded, including GSE6631, GSE13601, GSE23036, GSE30784, and GSE107591 (Table 1). Finally, 235 DEGs were identified with 103 up‐regulated and 132 down‐regulated DEGs after reprocessing for raw microarray data of expression files in the five GEO datasets. The top 20 significant differentially up‐regulated and down‐regulated genes from five GEO datasets are shown in Figure 1.

### 3.2 GO and KEGG analysis for DEGs

**FIGURE 2:**
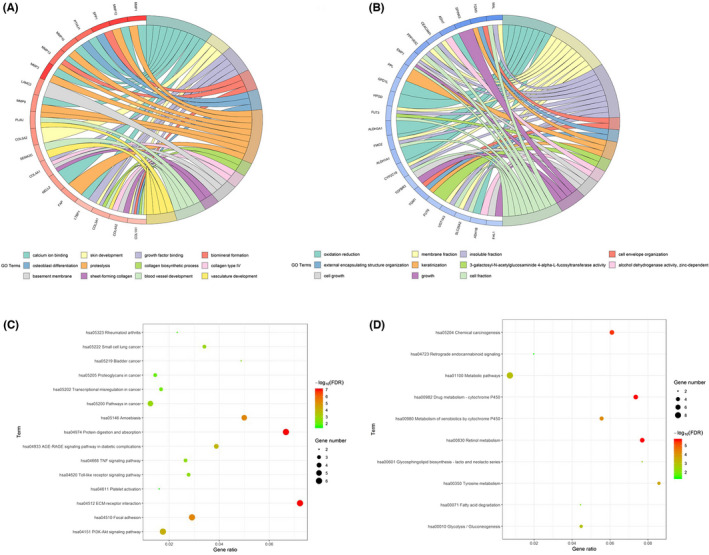
(A) GO enrichment analysis of up‐regulated DEGs. (B) GO enrichment of down‐regulated DEGs. (C) The bubble map of KEGG pathway analysis for up‐regulated DEGs. (D) The bubble map of KEGG pathway analysis for down‐regulated DEGs. DEGs, differentially expressed genes; GO, Gene Ontology; KEGG, Kyoto Encyclopedia of Genes and Genomes

The up‐ and down‐regulated DEGs were used for GO analysis by GOChord R package, respectively. The DEGs were significantly enriched in various GO categories of molecular function (MF), biological process (BP), and cellular component (CC). And the top 12 and 11 GO terms for up‐and down‐regulated DEGs are listed in Table S1. Based on the GOChord plotting function, for BP, the up‐regulated DEGs were significantly enriched in skin development (GO:0043588), biomineral formation (GO:0031214), osteoblast differentiation (GO:0001649), proteolysis (GO:0006508), collagen biosynthetic process (GO:0032964), blood vessel development (GO:0001568), and vasculature development (GO:0001944) and the down‐regulated DEGs were significantly enriched in oxidation reduction (GO:0055114), cell envelope organization (GO:0043163), external encapsulating structure organization (GO:0045229), keratinization (GO:0031424), cell growth (GO:0016049), and growth (GO:0040007) (Figure 2A,B). For MF, the DEGs with up‐regulation were significantly enriched in calcium ion binding (GO:0005509) and growth factor binding (GO:0019838) and the DEGs with down‐regulation were significantly enriched in 3‐galactosyl‐N‐acetylglucosaminide 4‐alpha‐L‐fucosyltransferase activity (GO:0017060) and alcohol dehydrogenase activity, zinc‐dependent (GO:0004024) (Figure 2A,B). For CC, the DEGs with upregulation were significantly enriched in collagen type IV (GO:0005587), basement membrane (GO:0005604), sheet‐forming collagen (GO:0030935) and the DEGs with downregulation were significantly enriched in membrane fraction (GO:0005624), insoluble fraction (GO:0005626), and cell fraction (GO:0000267) (Figure 2A,B). KEGG analysis was performed in order to know the biological pathways of the DEGs. The DEGs with up‐regulation were most significantly enriched in extracellular matrix (ECM)‐receptor interaction (hsa04512), Protein digestion and absorption (hsa04974) and Amoebiasis (hsa05146) (Figure 2C and Table S2) and the DEGs with down‐regulation were most significantly enriched in Retinol metabolism (hsa00830), Drug metabolism‐cytochrome P450 (hsa00982), and Chemical carcinogenesis (hsa05204) (Figure 2D and Table S2).

### 3.3 Construction of PPI network and module analysis

**FIGURE 3:**
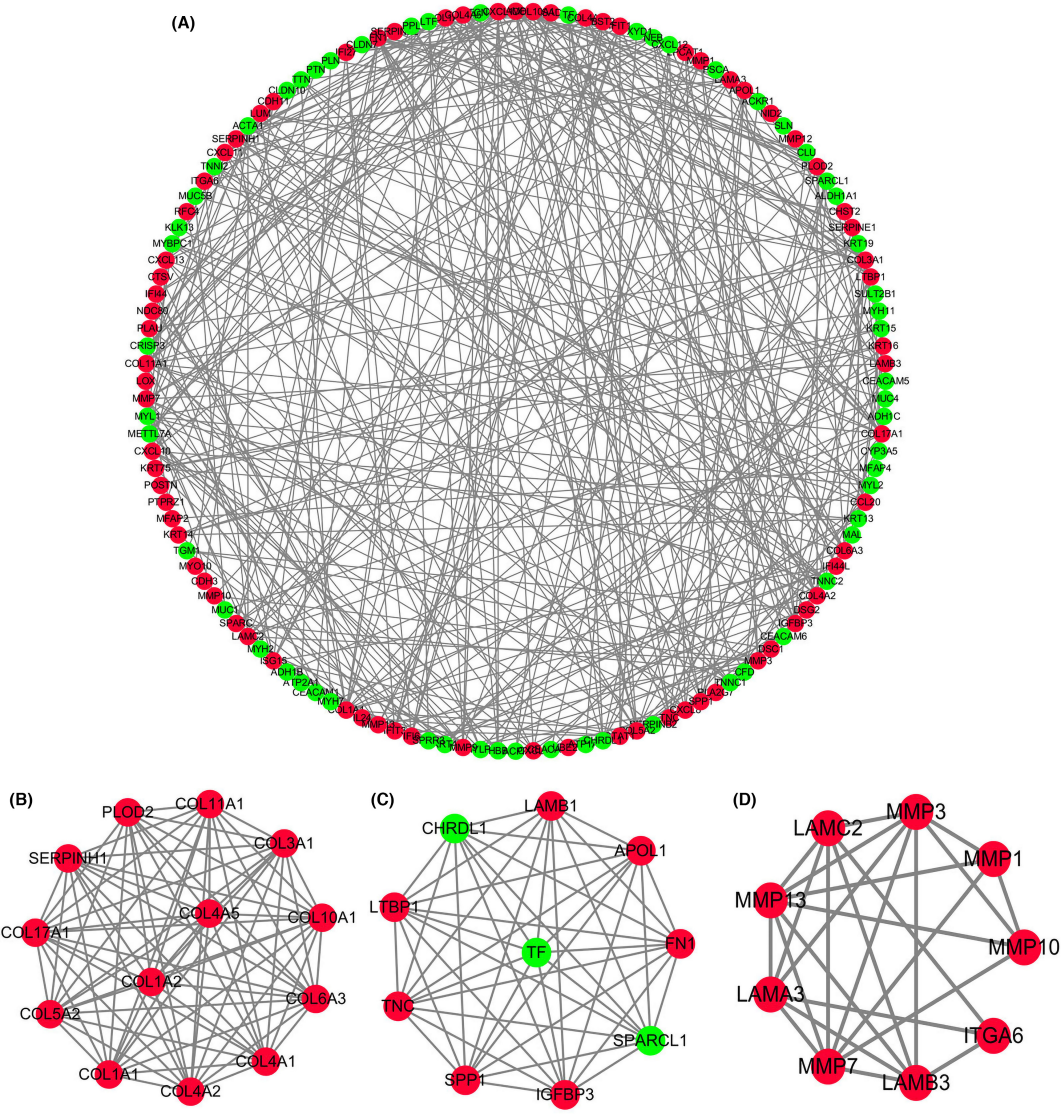
Protein‐protein interaction network of DEGs using STRING. (A) Entire PPI network. (B–D) PPI network of module 1, 2, and 3, respectively. DEGs, differentially expressed genes; PPI, protein‐protein interaction; STRING, Search Tool for the Retrieval of Interacting Genes

Interaction networks for different proteins will help us to systematically understand the molecular mechanisms in the development of HNSCC. PPI networks were constructed using STRING database and all the nodes without connections will be removed from PPI network. The genes with the most highly connected cluster were extracted by MCODE plug‐in in Cytoscape after PPI networks were analyzed. The DMNC and MCC algorithms of Cytoscape were used to identify hub genes. The top 10 hub genes based on the two methods were screened, and six mutual hub genes were isolated, including *COL4A1*, *MMP1*, *PLAU*, *RBP1*, *SEMA3C*, and *COL4A2* (Figure 3). These six genes all significantly up‐regulated in HNSCC samples compared with normal samples were used for further analysis.

### 3.4 Hub genes analysis

**FIGURE 4:**
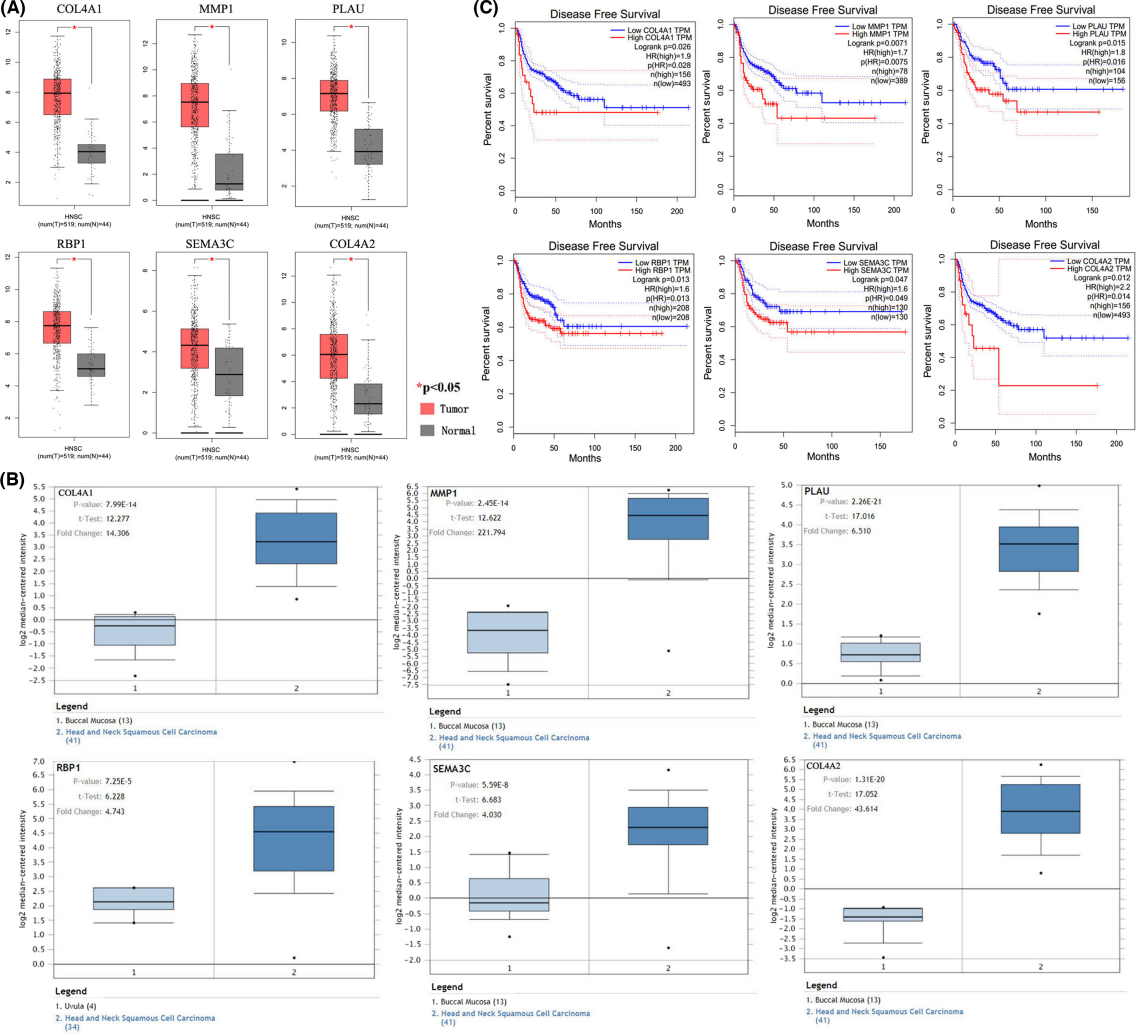
Validation of expression levels and survival analysis of hub genes. (A) Box plots of expression data from TCGA Database. (B) Expression levels based on Oncomine data. (C) Survival analysis based on TCGA and GEPIA. TCGA, The Cancer Genome Atlas

In order to better know the expression pattern of the hub genes, the expression of the hub genes was analyzed using the TCGA database which contains 519 HNSCC tumor samples and 44 normal samples. As Figure 4A showed, the expression of all the hub genes showed a significantly increase in HNSCC tumor samples when compared with normal samples (*p* < 0.05). These results were also confirmed by the expression changes in Oncomine database for *COL4A1* (*p* = 9.99E‐14), *MMP1* (*p* = 2.45E‐14), *PLAU* (*p* = 2.26E‐21), *RBP1* (*p* = 7.25E‐5), *SEMA3C* (*p* = 5.59E‐8), and *COL4A2* (*p* = 1.31E‐20) (Figure 4B). In addition, the disease‐free survival rate of *COL4A1*, *MMP1*, *PLAU*, *RBP1*, *SEMA3C*, and *COL4A2* were analyzed using Kaplan‐Meier curve. As Figure 4C shows, HNSCC patients with high transcripts per million for all the hub genes showed poorer survival rates. These results demonstrated that the six hub genes may play crucial roles in the development of HNSCC.

### 3.5 Hub genes were upregulated in HNSCCs

**FIGURE 5:**
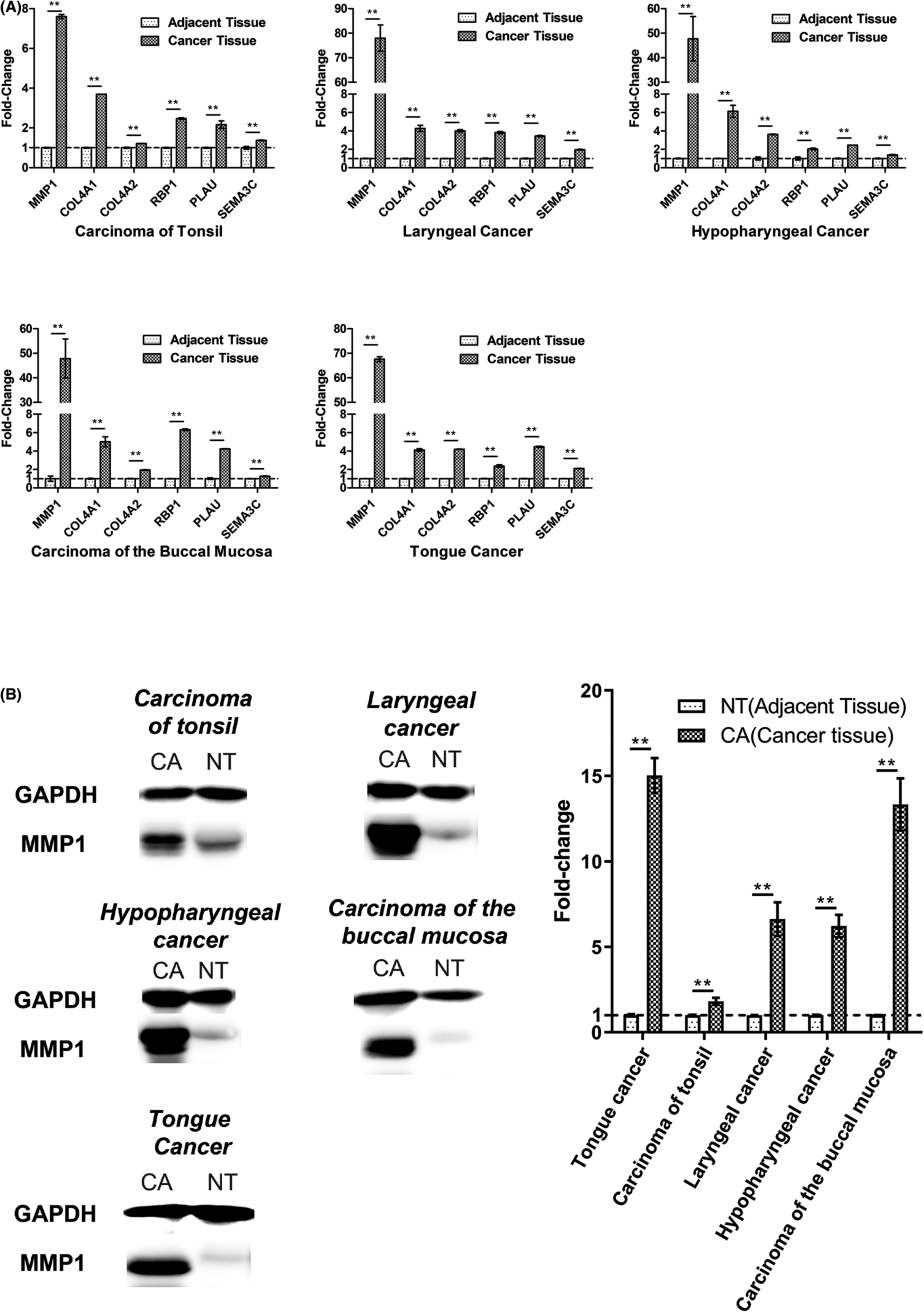
Expression of hub genes in clinical samples (*n* = 3). (A) mRNA expression of *MMP1*, *COL4A1*, *COL4A2*, *RBP1*, *PLAU*, and *SEMA3C*. (B) Protein expression of MMP1. CA, cancer tissues; NT, adjacent normal tissues. **, *p* < 0.01

We detected the expression of these six hub genes in carcinoma of tonsil, laryngeal cancer, hypopharyngeal cancer, carcinoma of the buccal mucosa, and tongue cancer. These genes were all expressed higher in these cancer tissues than their adjacent tissues (Figure 5A), and the fold‐changes in these genes are shown in Table 2. Among the six hub genes, the difference in *MMP1* expression between cancer tissues and adjacent tissues was the highest. Thus, we further detected the protein expression of *MMP1* in these clinical samples. The WB result showed that MMP1 protein expressed much higher in cancer tissues than adjacent normal tissues (Figure 5B). Fold‐changes in tongue cancer, carcinoma of tonsil, laryngeal cancer, hypopharyngeal cancer, and carcinoma of the buccal mucosa were 15.030 ± 1.019, 1.812 ± 0.202, 6.626 ± 0.979, 6.216 ± 0.655 and 13.333 ± 1.527.

**TABLE 2:** Fold‐change of hub genes in clinical HNSCC tissues (mean ± SD)

	*MMP1*	*COL4A1*	*COL4A2*	*RBP1*	*PLAU*	*SEMA3C*
Carcinoma of tonsil	7.603 ± 0.110	3.697 ± 0.000	1.217 ± 0.005	2.473 ± 0.034	2.168 ± 0.191	1.376 ± 0.022
Laryngeal cancer	77.996 ± 5.322	4.274 ± 0.325	4.019 ± 0.122	3.827 ± 0.121	3.457 ± 0.070	1.959 ± 0.049
Hypopharyngeal cancer	47.726 ± 9.041	6.127 ± 0.656	3.621 ± 0.038	2.037 ± 0.087	2.455 ± 0.010	1.391 ± 0.042
Carcinoma of the buccal mucosa	47.880 ± 7.938	5.020 ± 0.538	1.940 ± 0.027	6.320 ± 0.088	4.238 ± 0.017	1.257 ± 0.040
Tongue cancer	67.647 ± 0.979	4.112 ± 0.152	4.209 ± 0.017	2.375 ± 0.136	4.469 ± 0.065	2.094 ± 0.022

### 3.6 Knockdown of MMP1 inhibited cell growth, migration, and phosphorylation of AKT in vitro

**FIGURE 6:**
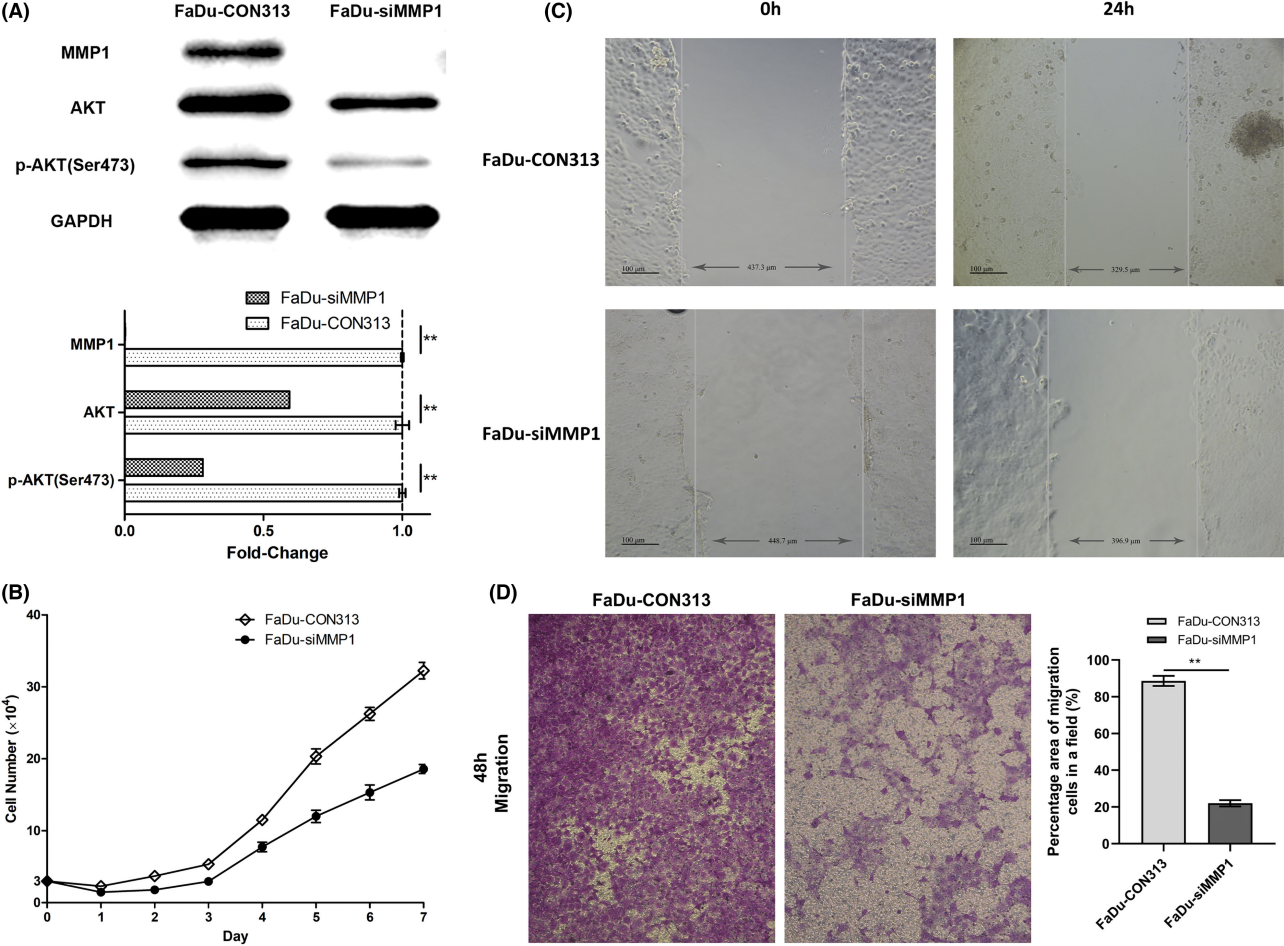
Silence of *MMP1* in vitro. (A) Protein expression of MMP1, AKT and p‐AKT (Ser473). (B) Cell growth curve (*n* = 3). (C) Wound healing assay (*n* = 3). (D) Transwell assay (*n* = 3). Images were captured under 10 × 10 microscope. **, *p* < 0.01

We used siMMP1 lentivirus and controlling lentivirus to stably transfect FaDu cells. The WB results demonstrated that infection of siMMP1 lentivirus can decrease the expression of MMP1 (Figure 6A). The fold‐change of MMP1 in FaDu‐siMMP1 was 0.002 ± 0.001.

Subsequently, we detected cell growth of FaDu‐siMMP1 and FaDu‐CON313 as control for consecutive 7 days. The cell growth curve indicated that after knockdown of MMP1, cells grew much more slowly during the whole week (Figure 6B). We then conducted wound‐healing assay and transwell assay. The wound‐healing assay showed that FaDu‐siMMP1 crept in a shorter distance than FaDu‐CON313 (Figure 6C). The transwell assay revealed that migrated cells of FaDu‐siMMP1 were less than FaDu‐CON313 (Figure 6D). The percentage area of migrated cells was 22.03 ± 1.71% in a field after silencing MMP1, while that of the control group was 88.60 ± 2.73%. The absence of MMP1 can attenuate cell growth and migration.

We conducted WB to identify the pathway affected by the knockdown of MMP1. AKT and p‐AKT (Ser473) both expressed lower in FaDu‐siMMP1 than FaDu‐CON313. But the expression of p‐AKT (Ser473) was more inhibited than total AKT in FaDu‐siMMP1 cells (Figure 6A). The fold‐changes of AKT and p‐AKT (Ser473) in FaDu‐siMMP1 were 0.594 ± 0.0001 and 0.282 ± 0.00009. MMP1 could increase the expression and phosphorylation of AKT to activate AKT pathway.

### 3.7 AKT inhibition attenuated cell growth and migration

**FIGURE 7:**
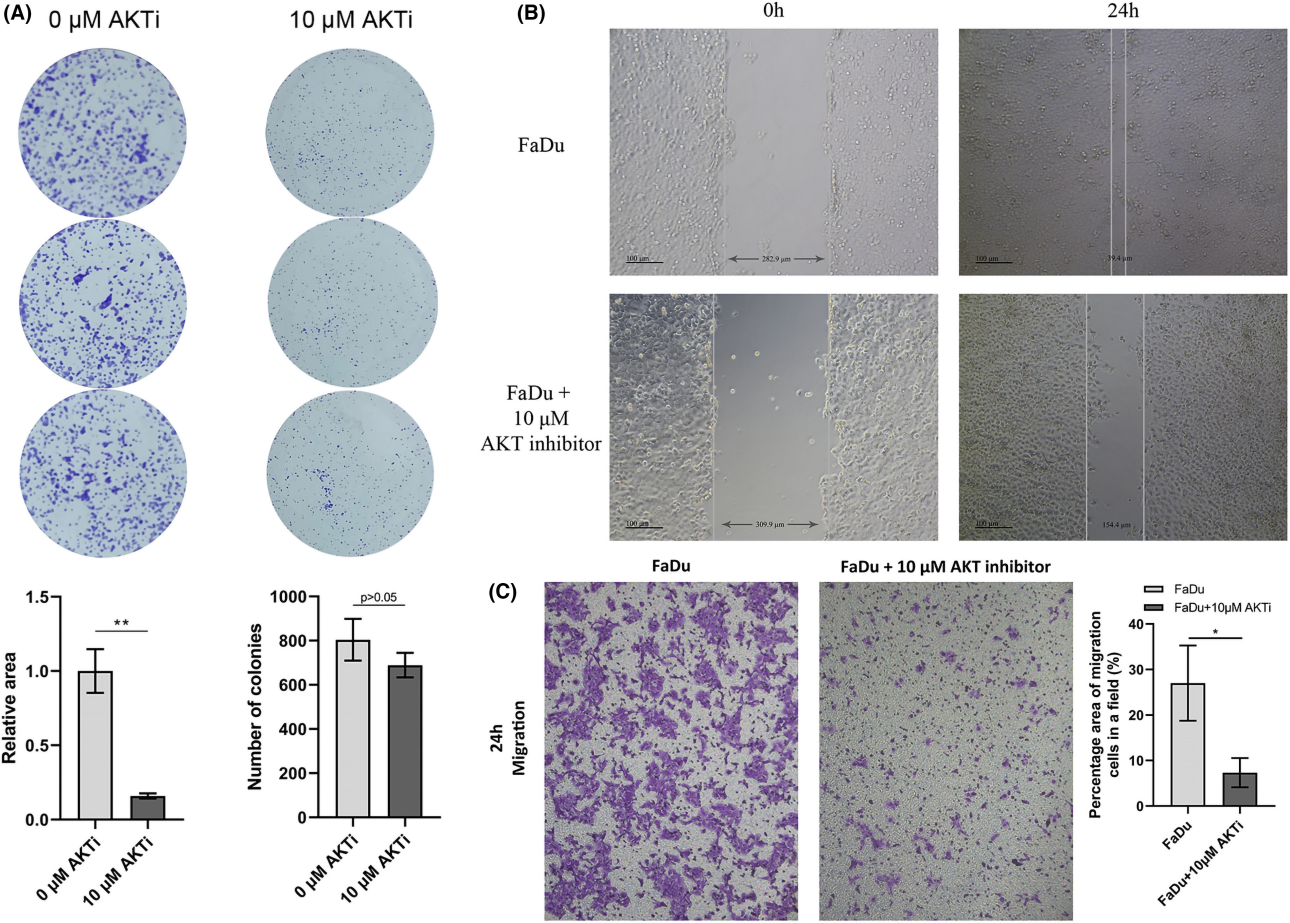
Inhibition of AKT in vitro. (A) Colony formation assay (*n* = 3). (B) Wound healing assay (*n* = 3). (C) Transwell assay (*n* = 3). Images were captured under 10 × 10 microscope. *, *p* < 0.05; **, *p* < 0.01

We then explored the relationship between AKT inhibition and the reduced ability of proliferation and migration. For colony formation assay, the area of colonies in the experimental group was significantly bigger than that in the control (Figure 7A). The area of experimental group was 15.96 ± 1.73% of the area of control group. But the difference in colony numbers between the two groups was not statistically significant. The wound‐healing assay showed that the presence of AKT inhibitor decreased the creeping distance of FaDu cells (Figure 7B). Similarly, the transwell assay also demonstrated that inhibition of AKT attenuated the ability of migration. The area of FaDu cells passed through pores accounted for 7.33 ± 3.21% in a field after AKT being inhibited, while the area of migrated untreated FaDu cells accounted for 27.01 ± 8.28% (Figure 7C). These results indicated that AKT inhibition can also attenuate tumor progression as same as silencing MMP1. MMP1 activated AKT pathway to promote proliferation and migration of hypopharyngeal cancer cells.

### 3.8 Absence of MMP1 decreased tumor migration but not growth in vivo

**FIGURE 8:**
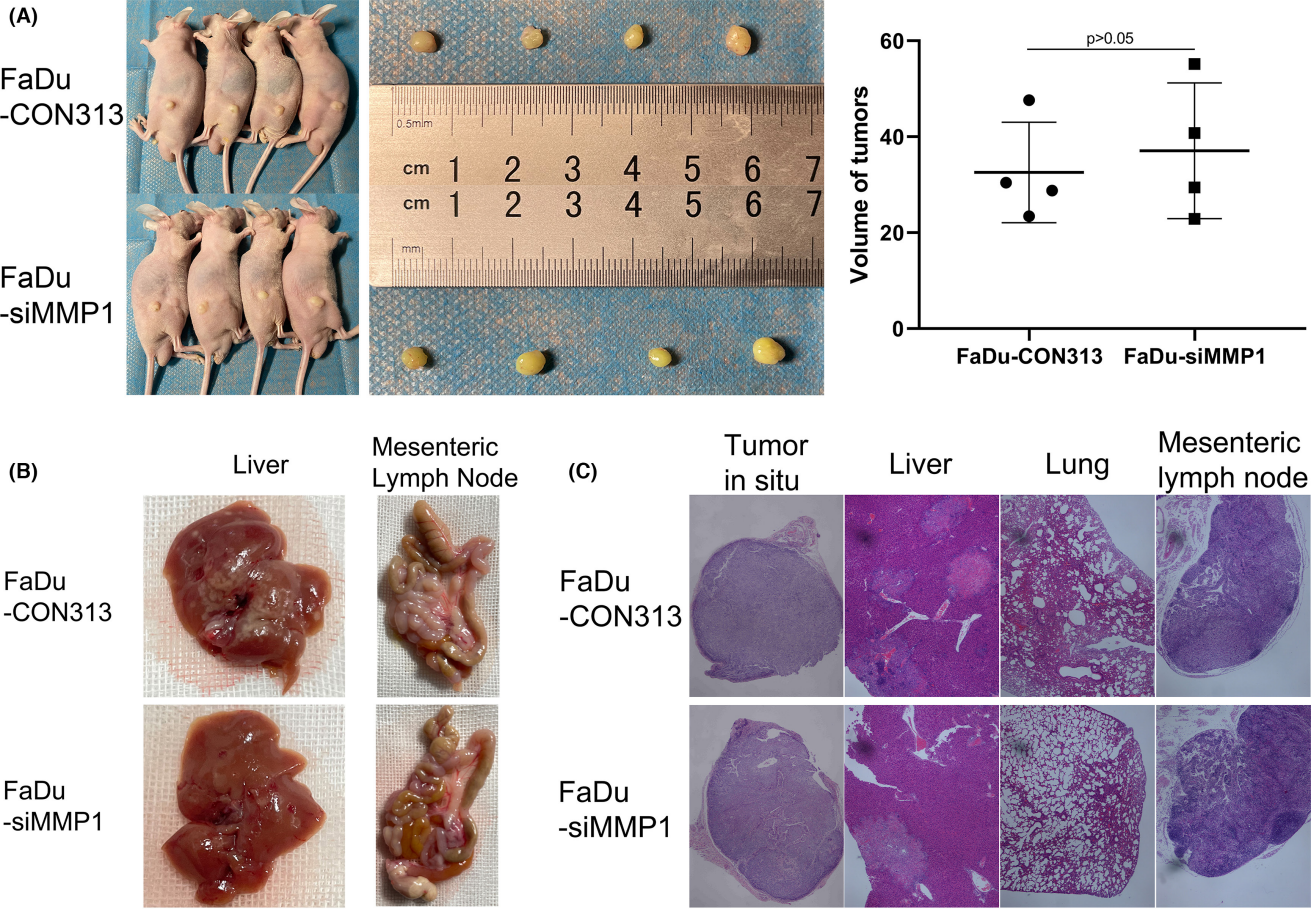
Silence of *MMP1* in vivo. (A) Macroscopic view of tumors in situ. Volume = length × width^2^/2. (*n* = 4). (B) Macroscopic view of livers and swelling mesenteric lymph nodes. (C) HE staining. Slices were observed under 4 × 10 microscope

We conducted xenograft tumor models to explore the function of MMP1 in vivo. The subdermal tumors derived from FaDu‐siMMP1 were not statistically different from tumors in the FaDu‐CON313 group (Figure 8A). The volume of FaDu‐CON313 group and FaDu‐siMMP1 group was 32.56 ± 10.48 and 37.07 ± 14.16 mm^3^. However, the liver metastasis in the FaDu‐CON313 group was more severe than that in the FaDu‐siMMP1 group. There were much more macroscopical gray metastases in the livers of FaDu‐CON313 group (Figure 8B). But the swelling mesenteric lymph nodes could be observed both in the FaDu‐CON313 group and FaDu‐siMMP1 group (Figure 8B). To deeper understand the situation of multiple organ metastases, we conducted HE staining of lungs, livers, and swelling mesenteric lymph nodes in both groups. Shown in Figure 8C, metastatic necroses were more in FaDu‐CON313 group compared to FaDu‐siMMP1 group in livers. But metastasis was not found in lungs and swelling mesenteric lymph nodes.

## 4 DISCUSSION

HNSCC is a malignant tumor that could lead to high mortality rate with an extremely poor 5‐year survival rate. Although amounts of molecular biomarkers have been identified, the biomarkers which were truly used for the diagnosis, prognosis in HNSCC are still scarce. In the present study, five high‐quality GEO datasets were selected to excavate the hub genes and the biological pathways of the hub genes involved in HNSCC by integrated bioinformatics analysis. Finally, 103 up‐regulated and 132 down‐regulated genes were identified. Six hub genes were screened out which showed dramatically increased expression level in HNSCC samples, including *COL4A1*, *MMP1*, *PLAU*, *RBP1*, *SEMA3C*, and *COL4A2*. These hub genes all showed worse disease free survival with higher expression level. Besides, the expression of *MMP1* was found to be the most significantly differential. Our study demonstrated that *MMP1* attenuated cell growth, migration, and phosphorylation of AKT. These results indicated that these hub genes, especially *MMP1*, might participate in regulating the progression of HNSCC and could be potential molecular biomarkers in the precise diagnosis of HNSCC.

The GO analysis revealed that DEGs were significantly enriched in MF of calcium ion binding (GO:0005509) and BP of skin development (GO:0043588). Yang et al demonstrated that the DEGs were significantly enriched in protein binding and calcium ion binding by meta‐analysis of DEGs in osteosarcoma based on microarray data.^20^ However, there is no study related to HNSCC. In this study, the expression level of the genes related to calcium ion binding in patients with HNSCC, such as *PTHLH*, *MMP10*, *LTBP1*, *MMP9*, *NELL2*, *MMP3*, *MMP13*, *MMP12*, and *MMP1* were significantly altered, indicating that these genes might be crucial to HNSCC development related to calcium ion binding. Meanwhile, KEGG pathway analysis revealed that the upregulated DEGs were significantly enriched in ECM‐receptor interaction (has04512) and Protein digestion and absorptiohashsa04974). Previous studies have shown that ECM remodeling not only promotes cancer development but also indicates poor prognosis in HNSCC patients.^21^ The GO and KEGG pathways analysis of the DEGs provide a solid foundation for revealing the BPs and mechanisms involved in the development of HNSCC.

In line with the GO and KEGG analysis, six genes were considered to be hub genes in PPI network, and their expression characteristics were also verified by TCGA database. So far, increasing numbers of studies have demonstrated that the expression of collagen family members, such as COL1A1 and COL1A2, was abnormal in several cancers, such as breast and lung cancers.^22^, ^23^, ^24^ *PLAU* has already been demonstrated to be involved in metastasis and immune escape for different tumors.^25^ Recent studies showed that *PLAU* may act as a biomarker of HNSCC based on integrated bioinformatics analysis.^9^, ^10^ Vaitkienė et al identified that *SEMA3C* had a potential as a prognostic marker for glioma, breast, and oral neoplasia, respectively.^26^, ^27^ Recently, Hui and Liu et al considered that *SEMA3C* was associated with poor prognosis in cervical cancer and might act as a therapeutic target in prostate and other cancers.^28^, ^29^ Chen et al demonstrated that the expression of *RBP1* is up‐regulated in tongue squamous cell carcinoma.^30^

Many studies have proved that *MMP1* is associated with tumor progression in different kinds of cancers. For example, *MMP1* was highly expressed in colorectal cancer tissues and its high expression was related to lymphatic metastasis and TNM stage. The experiments in vitro and in vivo demonstrated that knockdown of *MMP1* attenuated tumor growth and migration.^31^ In esophageal squamous cell cancer, *MMP1* was expressed higher in tumor tissues than their adjacent normal tissues. Its high expression was associated with lymph node metastasis, microvessel density, and advanced TNM stage. The experiments in vitro and in vivo also proved that overexpression of *MMP1* promoted cell growth, migration, and colony formation.^32^ *MMP1* regulated by ETV4 was also found to promote cell migration and invasion in nonsmall‐cell lung cancer.^33^ In triple‐negative breast cancer, *MMP1* regulated by YB‐1 can promote invasion.^34^ In addition, *MMP1* was proved to activate PI3K‐AKT pathway and epithelial‐mesenchymal transition.^31^, ^32^ In cervical squamous cell carcinoma, high expressed *MMP1* was associated with poor prognosis and negatively related to the amount of T cells and macrophages infiltration.^35^ *MMP1* might be a biomarker for immunotherapy and prognostic judgment in cervical squamous cell carcinoma. In HNSCC, there were several reports about *MMP1* and its effects on tumor progress. Lallemant et al reported that *MMP1* shows high sensitivity and specificity for serving as a diagnostic marker in HNSCC.^36^ A research about human oral squamous cell carcinoma showed that *MMP1* was expressed higher in tumor than normal tissue and its higher expression was positively correlated with a shorter overall survival rate.^37^ Wang et al also found that *MMP1* 3’UTR can sponge miR‐188‐5p to facilitate the proliferation and migration of human oral squamous cell carcinoma.^38^ Consistently, our results showed that *MMP1* was significantly increased in HNSCCs and its high expression was positively correlated with poor prognosis. However, few research has focused on *MMP1* expression and effect in hypopharyngeal cancer specifically. Our experiment in vitro showed that the absence of *MMP1* in hypopharyngeal cancer cells attenuated cell growth and migration. And *MMP1* was also found to promote liver metastatic necrosis of hypopharyngeal cancer in vivo. Interestingly, our experiments in vivo did not show slowly growing tumor after decreasing *MMP1* expression. Ignoring the individual differences of the nude mice, we speculated that cells proliferated more in the control group than the silencing *MMP1* group, but the increased cells contributed to liver metastasis instead of growing in situ. In this study, we verified that *MMP1* promoted tumor progress and could serve as a biomarker in HNSCC, especially in hypopharyngeal cancer.

## 5 CONCLUSION

In the present study, we have identified six hub genes involved in HNSCC using integrated bioinformatics analysis (*COL4A1*, *MMP1*, *PLAU*, *RBP1*, *SEMA3C*, and *COL4A2*). These hub genes all showed worse disease‐free survival with higher expression in an online database. Then, we verified that these hub genes were expressed higher in our clinical samples of HNSCC. We demonstrated that *MMP1* can facilitate cell growth and migration in vitro by phosphorylating the AKT pathway. Furthermore, we proved that *MMP1* promotes liver metastasis in vivo. Thus, *MMP1* might serve as a biomarker for HNSCC.

## CONFLICT OF INTEREST

The authors declare that they have no competing interests.

## AUTHOR CONTRIBUTIONS

Gehou Zhang, Tieqi Li, and Yexun Song: designed and conducted the research, analyzed the data, and drafted the manuscript. Guolin Tan: designed the research and revised the article. Qian Liu and Kai Wang: conducted the research and drafted the manuscript. Jingang Ai and Zheng Zhou: analyzed the data and revised the article. Wei Li: designed and conducted the research, drafted the manuscript, and revised the article.

## ETHICS APPROVAL AND CONSENT TO PARTICIPATE

Collection and usage of clinical specimens was approved by the ethics committee of the third Xiangya hospital of Central South University (No.2020‐S133). The animal experiment was conducted in the department of laboratory animals of Central South University and approved by ethics committee (No.2021sydw0172).

## CONSENT FOR PUBLICATION

Not applicable.

## Supporting information


Table S1

Table S2

## Data Availability

The datasets analyzed during the current study are available in the GEO, https://www.ncbi.nlm.nih.gov/geo/.
