# Supplementary material for: Identity of MMP1 and its effects on tumor progression in head and neck squamous cell carcinoma
Source: Cancer Med. 2022 Apr 14;11(12):2516–30. doi: 10.1002/cam4.4623 (PMC9189457; doi:10.1002/cam4.4623)
Supplement: Supplementary file 1 — Table S1 [file CAM4-11-2516-s001.docx]

**Supplementary Table Ⅰ. Go analysis for up and down-regulated DEGs, respectively.**

| **Category** | **ID** | **Term** | **Genes** | **Adjp-value** |
| --- | --- | --- | --- | --- |
| MF | GO:0005509 | calcium ion binding | *PTHLH, MMP10, LTBP1, MMP9, NELL2, MMP3, MMP13, MMP12, MMP1* | 0.001393 |
| BP | GO:0043588 | skin development | *COL3A1, COL1A1, COL5A2* | 0.002115 |
| MF | GO:0019838 | growth factor binding | *LTBP1, COL4A1, COL3A1, COL1A1* | 0.00215 |
| BP | GO:0031214 | biomineral formation | *COL1A1, MMP13, SPP1* | 0.003429 |
| BP | GO:0001649 | osteoblast differentiation | *PTHLH, COL1A1, SPP1* | 0.0044 |
| BP | GO:0006508 | proteolysis | *MMP10, MMP9, FAP, MMP3, MMP13, MMP12, MMP1, PLAU* | 0.010234 |
| BP | GO:0032964 | collagen biosynthetic process | *COL3A1, COL1A1* | 0.011773 |
| CC | GO:0005587 | collagen type IV | *COL4A2, COL4A1* | 0.015857 |
| CC | GO:0005604 | basement membrane | *COL4A2, COL4A1, LAMC2* | 0.018179 |
| CC | GO:0030935 | sheet-forming collagen | *COL4A2, COL4A1* | 0.018476 |
| BP | GO:0001568 | blood vessel development | *COL3A1, SEMA3C, COL1A1, PLAU* | 0.019769 |
| BP | GO:0001944 | vasculature development | *COL3A1, SEMA3C, COL1A1, PLAU* | 0.021062 |
| **Category** | **ID** | **Term** | **Genes** | **Adjp-value** |
| BP | GO:0055114 | oxidation reduction | *ALDH1A1, GPD1L, CYP2C18, FMO2, ADH1B, ADH7, HPGD, ALDH3A1* | 0.001073 |
| CC | GO:0005624 | membrane fraction | *PPP1R3C, UGT1A3, CYP2C18, FMO2, FUT3, MAL, SLC26A2, EMP1, CEACAM1* | 0.002223 |
| CC | GO:0005626 | insoluble fraction | *PPP1R3C, UGT1A3, CYP2C18, FMO2, FUT3, MAL, SLC26A2, EMP1, CEACAM1* | 0.002792 |
| BP | GO:0043163 | cell envelope organization | *TGM1, TGM3* | 0.005168 |
| BP | GO:0045229 | external encapsulating structure organization | *TGM1, TGM3* | 0.005168 |
| BP | GO:0031424 | keratinization | *PPL, TGM1, TGM3* | 0.005494 |
| MF | GO:0017060 | 3-galactosyl-N-acetylglucosaminide 4-alpha-L-fucosyltransferase activity | *FUT6, FUT3* | 0.007607 |
| MF | GO:0004024 | alcohol dehydrogenase activity, zinc-dependent | *ADH1B, ADH7* | 0.01013 |
| BP | GO:0016049 | cell growth | *FHL1, TGFBR3, EMP1* | 0.010815 |
| BP | GO:0040007 | growth | *FHL1, TGFBR3, SPINK5, EMP1* | 0.0116 |
| CC | GO:0000267 | cell fraction | *PPP1R3C, UGT1A3, CYP2C18, FMO2, FUT3, MAL, SLC26A2, EMP1, CEACAM1* | 0.012785 |
