# Supplementary material for: Identity of MMP1 and its effects on tumor progression in head and neck squamous cell carcinoma
Source: Cancer Med. 2022 Apr 14;11(12):2516–30. doi: 10.1002/cam4.4623 (PMC9189457; doi:10.1002/cam4.4623)
Supplement: Supplementary file 2 — Table S2 [file CAM4-11-2516-s002.docx]

**Supplementary Table Ⅱ. KEGG analysis for up and down-regulated DEGs, respectively.**

| **Term** | **Count** | | **Ratio** | **FDR** |
| --- | --- | --- | --- | --- |
| hsa04512 ECM-receptor interaction | | 6 | 0.072289 | 9.23E-08 |
| hsa04974 Protein digestion and absorption | | 6 | 0.066667 | 9.23E-08 |
| hsa05146 Amoebiasis | | 5 | 0.05 | 4.98E-06 |
| hsa04510 Focal adhesion | | 6 | 0.029126 | 5.17E-06 |
| hsa04151 PI3K-Akt signaling pathway | | 6 | 0.017493 | 7.32E-05 |
| hsa04933 AGE-RAGE signaling pathway in diabetic complications | | 4 | 0.038835 | 0.000101 |
| hsa05200 Pathways in cancer | | 5 | 0.012531 | 0.001346 |
| hsa05222 Small cell lung cancer | | 3 | 0.034091 | 0.001346 |
| hsa04620 Toll-like receptor signaling pathway | | 3 | 0.027778 | 0.00214 |
| hsa04668 TNF signaling pathway | | 3 | 0.026549 | 0.00219 |
| hsa05219 Bladder cancer | | 2 | 0.04878 | 0.005498 |
| hsa05202 Transcriptional misregulation in cancer | | 3 | 0.01676 | 0.006657 |
| hsa05205 Proteoglycans in cancer | | 3 | 0.014423 | 0.009321 |
| hsa05323 Rheumatoid arthritis | | 2 | 0.023256 | 0.017568 |
| hsa04611 Platelet activation | | 2 | 0.016 | 0.033064 |
| **Term** | | **Count** | **Ratio** | **FDR** |
| hsa00830 Retinol metabolism | | 5 | 0.076923 | 2.30E-06 |
| hsa00982 Drug metabolism-cytochrome P450 | | 5 | 0.073529 | 2.30E-06 |
| hsa05204 Chemical carcinogenesis | | 5 | 0.060976 | 3.70E-06 |
| hsa00980 Metabolism of xenobiotics by cytochrome P450 | | 4 | 0.055556 | 6.64E-05 |
| hsa00350 Tyrosine metabolism | | 3 | 0.085714 | 0.000242 |
| hsa01100 Metabolic pathways | | 9 | 0.007258 | 0.000916 |
| hsa00010 Glycolysis / Gluconeogenesis | | 3 | 0.044776 | 0.00108 |
| hsa00601 Glycosphingolipid biosynthesis-lacto and neolacto series | | 2 | 0.076923 | 0.004416 |
| hsa00071 Fatty acid degradation | | 2 | 0.044444 | 0.011009 |
| hsa04723 Retrograde endocannabinoid signaling | | 2 | 0.019802 | 0.045455 |
